# The Effects of Ellagic Acid on Experimental Corrosive Esophageal Burn Injury

**DOI:** 10.3390/cimb46020102

**Published:** 2024-02-16

**Authors:** Dilek Aygün Keşim, Fırat Aşır, Hayat Ayaz, Tuğcan Korak

**Affiliations:** 1 Department of Physiology, Medical Faculty, Dicle University, Diyarbakır 21280, Turkey; dilekaygunkesim@gmail.com; 2 Department of Histology and Embryology, Medical Faculty, Dicle University, Diyarbakır 21280, Turkey; ayazhayat44@gmail.com; 3 Department of Medical Biology, Medical Faculty, Kocaeli University, Kocaeli 41001, Turkey; tugcankorak@gmail.com

**Keywords:** ellagic acid, antioxidant, anti-inflammatory, corrosive substance, NaOH

## Abstract

This study aimed to investigate the antioxidant effect of Ellagic acid (EA) on wound healing in sodium hydroxide (NaOH)-induced corrosive esophageal burn injury. The interaction networks and functional annotations were conducted using Cytoscape software. A total of 24 Wistar albino rats were divided into control, corrosive esophageal burn (CEB) and CEB + EA groups. Burn injury was created by 20% NaOH and 30 mg/kg EA was per oral administered to rats. At the end of the 28-day experimental period, Malondialdehyde (MDA) content was measured. Esophageal tissue samples were processed for histological staining. The EA–target interaction network was revealed to be involved in regulating crucial cellular mechanisms for burn wound healing, with epidermal growth factor (EGF) identified as a central mediator. An increase in animal weight in the CEB + EA group was observed in the EA-treated group after CEB injury. Burn injury increased MDA content, but EA treatment decreased its level after CEB injury. Stenosis index, collagen degeneration, inflammation, fibrosis and necrosis levels were increased after CEB injury. EA treatment improved histopathology in the CEB + EA group compared to the CEB group. The expression of EGF was decreased in the CEB group but upregulated in the EA-treated group, suggesting a potential involvement of EA in cellular processes and tissue regeneration. EA, through its antioxidative and tissue regenerative properties, significantly contributes to alleviating the adverse effects of CEB injury, promoting wound healing.

## 1. Introduction

Alkaline corrosive substance exposure is a serious health problem that causes mortality and morbidity in low- and middle-income countries [1]. Moreover, 80% of cases occur accidentally in children under 5 years of age [2]. The degree of tissue damage varies depending on the pH level, density, form, exposure duration and amount of the corrosive agent exposed [3]. The ingestion of alkaline corrosive substances causes tissue liquefaction necrosis, vascular thrombosis and lipid hydrolysis in the esophagus [4]. As a result, fibrotic stricture developed, leading to dysphagia, malnutrition and growth retardation in children [5]. The management of post-corrosive esophageal strictures is a major clinical issue. Urgent treatment is to limit the burn area with neutralizing chemical drugs [6]. Corticosteroids, antibiotics and surgical intervention are used to prevent stricture formation; however, some patients may not completely recover [7]. To prevent stricture formation, no exact preparation is available. Some pre-clinical studies investigated the potential curative roles of fluorouracil, octreotide, cytokines and antioxidant agents [8,9].

Ellagic acid (EA), an antioxidant and anti-inflammatory, can prevent stenosis and stricture formation, be used in corrosive esophageal burn (CEB) injury and may alleviate the pathological symptoms of the injury. EA is a polyphenolic compound found in pomegranates, strawberries, raspberries, cherries, cranberries, blackberries, red grapes and walnuts. Studies reported that EA has antitumor, antioxidant, antiviral, anti-fibrosis and antimicrobial effects and antioxidant activities. EA scavenges against reactive oxygen and protects cells against oxidative damage due to the hydroxyl group in its structure [10,11]. In an experimental burn study, EA prevented polymorphonuclear cell infiltration and collagen fiber deposition and induced re-epithelialization [12]. Mo et al. studied [13] the role of EA with its antioxidant properties on rat dermal wounds. They found that EA increased the tensile strength of wound incision, significantly reduced wound area and induced epithelization via promoting collagen synthesis and anti-inflammation pathway. The biopharmaceutical properties of EA were also investigated. Tavares et al. [14] loaded EA into chitosan/zein films and examined its role in wound bandaging. The authors found that EA load films could be used as a therapeutic option to treat skin regeneration and for the inhibition of cutaneous–mucous infections. A similar study performed by Zhang et al. [15] revealed that EA could be used as a cross-linker in hydrogel dressers for wound healing. The authors claimed that wound dressing was enforced with antioxidant, antibacterial and anti-inflammatory activities of EA, and results showed that wound dressing reduced the infected wound area in rats and promoted angiogenesis and collagen deposition. All these studies suggest a potential role of EA in esophageal burn injury due to its tissue regeneration.

The recognition of targets for compounds like EA involves the utilization of diverse computational methods such as proteochemometrics modeling, molecular dynamic/free energy perturbation, ligand-based interaction fingerprints and docking virtual screening. Furthermore, the exploration of target identification can be conducted through network-based drug discovery [16]. Within this field, the Search Tool for Interactions of Chemicals (STITCH)database integrates data concerning interactions from metabolic pathways, crystal structures, binding experiments and drug–target relationships, aiming to comprehend interactions between molecules and proteins. STITCH further enables the investigation of chemical relations in a network, encompassing associated binding proteins, thus offering a valuable tool for examining compound-related interaction networks from a broad perspective [17]

In the experimental studies, EA exerts its beneficial effects by regulating multiple pathways, including caspase-3; B-cell lymphoma 2 (Bcl-2), Bax (Bcl-2-associated X protein);; nuclear factor kappa B (NF-κB); telomerase, pro-inflammatory mediators, such as Interleukin (IL)-6, IL-1β and tumor necrosis factor (TNF)-α, cylins; metalloproteinases; Akt signaling and adhesion molecules, like E-selectin and E-cadherin [a, b]. Furthermore, EA has regulatory impacts on growth factors (GF) such asinsulin-like GF-2 (IGF-2), hepatocyte GF (HGF), transforming GF beta (TGF-β), platelet-derived (PDGF), vascular endothelial GF (VEGF) and fibroblast GF (FGF) [18,19]. Among these, EGF was employed in wound management and regenerative medicine since the late 1980s. Its widespread and enduring utilization is attributable to its exceptional tolerability and effectiveness [20].

Epidermal growth factor (EGF) is a mitogenic polypeptide that ensures the proliferation, migration and differentiation of fibroblast, endothelial and keratinocyte cells involved in wound healing [21]. EGF was extensively studied for its ability to accelerate wound closure, promote re-epithelialization and enhance tissue regeneration [22]. Preclinical studies suggested that Ellagic acid can potentiate the effects of growth factors by augmenting their binding affinity to growth factor receptors or prolonging their half-life in the extracellular milieu [23]. Brown et al. [24] conducted a clinical trial on patients with burn or reconstructive surgery. They topically applied silver sulfadiazine cream containing EGF and found that EGF accelerated the rate of epidermal regeneration in all patients. Similarly, Choi et al. [25] used biodegradable nanofibers containing EGF to treat diabetic ulcers in experimental rats. They found that wound-healing activities and EGF receptor expression were increased in the re-epithelialized tissue of animals.

During tissue regeneration, the role of EGF on wound healing is well known; however, no study shows the role of EA on wound healing via EGF. This study aimed to examine the effect of EA on experimental esophageal wound healing through its antioxidant properties.

## 2. Materials and Methods

### 2.1. Construction of Merged Ellagic Acid-Target PPI Network and Hub Gene Detection

El Ellagic acid target signaling pathway was constructed by using STITCH database in Cytoscape (Cytoscape Consortium, version: v3.10.1, San Diego, CA, USA) (maximum additional interactors: 40, confidence cutoff as the default option: 0.40, which represents the moderate level of confidence [26] (https://cytoscape.org/, accessed on: 15 October 2023). STRING: PubMed query was arranged as a data source that integrates PPI and relevant literature data for wound healing to create an extensive network (maximum additional interactors: 100, confidence cutoff: 0.40). Then, these two networks were merged in order to evaluate potential action mechanisms of EA in relation to the EGF and to examine the presence of potential pathways related to “burn wound healing” within its functional annotations. First and secondary neighbors were set as different circular layouts in a space-efficient manner. Functional annotations were performed for EGF and all nodes in the network. The functional enrichment analysis of these genes encompassed all categories within Cytoscape, incorporating closely associated mechanisms related to “burn wound healing” into the network. Moreover, we identified hub genes using the cytoHubba plug-in within Cytoscape. The top 20 central genes within the network were determined through CytoHubba using the Maximal Clique Centrality (MCC) algorithm, which demonstrated superior performance in accurately predicting essential proteins within the PPI network [27].

### 2.2. Animal Housing

Ethical approval for animal experiments was taken from the Animal Experiments Local Ethics Committee of Dicle University (date: 30 July 2022, approval no: 2022/27). Twenty-four Wistar albino rats (15–16 weeks, 250–270 g) were housed in cages at a temperature of 23 ± 2 °C, with a 12/12 h dark/light cycle. The rats had ad libitum access to water and standard pellets.

### 2.3. Ellagic Experimental Design

Following a 12 h fasting period, a foley catheter was used to measure distance between the mouth and cardio-esophageal junction under anesthesia. The catheter (2-way, 6FR, 5-15cc-Cod: CA100707, Hangzhou Fushan Medical Appliances Co, Hangzhou City, Zhejiang Province, 311301, China)was inserted into the stomach, and the catheter balloon was filled with 4 mm^3^ distilled water before being gently retracted, preventing gastrointestinal tract from burn injury.

**Figure 1:**
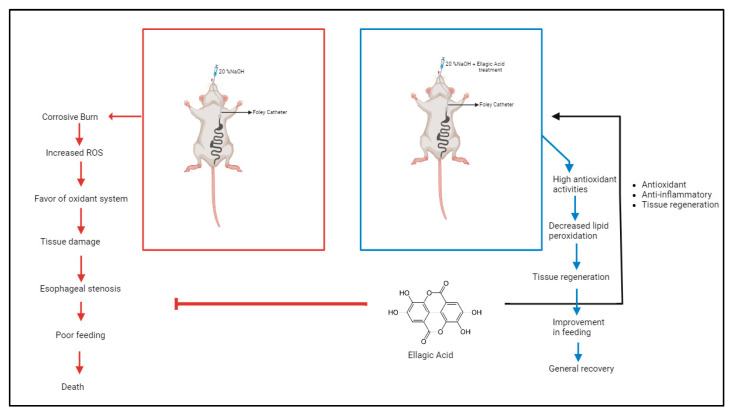
Summary of experimental steps (from Biorender.com).

Moreover, 20% NaOH solution was introduced through the catheter for 60 s and then aspirated. The wound area was thoroughly irrigated with distilled water, and the Foley catheter was retracted (Figure 1).

Initial weights of all animals were recorded before starting the experiment. The rats were categorized into three groups (8 rats per group).

1. Control Group: A total of 4 mL saline was administered to rats via Foley catheter. No further interventions were applied to the rats.
2. Corrosive Esophageal Burn (CEB) Group: Burn injury was induced, and no treatments were applied.
3. CEB + Ellagic Acid (CEB + EA): Burn injury was induced, and 30 mg/kg Ellagic acid (catalogn no: E2250, Merck KGaA, Darmstadt, Germany) was administered to rats per oral administered once a day for 28 days.

At the end of the experiment, the weights of all rats were recorded. All rats were sacrificed under anesthesia, and esophageal tissues were excised for further analysis.

### 2.4. Determination of Malondialdehyde (MDA)

Malondialdehyde (MDA) is the end product of lipid peroxidation and an indicator of oxidation stress. The thiobarbituric acid (TBA) method was used to evaluate the MDA content in esophageal tissues. Samples were weighed and homogenized in cold 0.5 mL 10% trichloroacetic acid + 4.5 mL 5% Trichloroacetic acid solution (TCA) (*w*/*v*). Samples were centrifuged at 3000× *g* rpm for 15 min, and supernatants were transferred to tubes with equal volume of 0.6% (*w*/*v*) TBA. The absorbance of the mixture was measured at 532 nm spectrum with a spectrophotometer. Results were presented as nmol/g protein using the molar extinction coefficient (155 mM^−1^ cm^−1^) [28].

### 2.5. Histopathological Tissue Preparation and Scoring

Esophageal samples were fixed in zinc–formalin and dehydrated through an increasing alcohol series (50%, 70%, 80%, 90%, 96%, absolute alcohol) and embedded in paraffin blocks [29]. Hematoxylin Eosin and Trichrome Masson dyes were applied to 5 μm thick sections and imaged with Zeiss Imager A2 light microscope. Semiquantitative scoring of histological parameters was calculated from 5 different areas of each specimen to evaluate burn injury. Submucosal collagen increase (SMI) scoring: none: 0, mild: 1, moderate: 2; muscularis mucosa damage (MMD) scoring: no: 0, yes: 1; tunica muscularis collagen deposition/damage (TMCD) scoring: none: 0, mild: 1, moderate: 2; Inflammation: none: 0, moderate: 1, high: 2, severe: 3; Fibrosis: none: 0, moderate: 1, high: 2, severe: 3; Necrosis: absent: 0, moderate: 1, high: 2, severe: 3. Stenosis Index: The luminal diameter of the esophagus and wall thickness was calculated. Stenosis index was defined as wall thickness/luminal diameter [30]. All histological scoring and measurements were performed by two blinded pathologists.

### 2.6. Immunohistochemical Staining

Esophageal sections were deparaffinized in xylene, hydrated through a descending series of ethanol and rinsed in distilled water. Sections were incubated with 3% hydrogen peroxide solution (cat# TA-015-HP, ThermoFisher, Fremont, CA, USA) for 20 min to prevent nonspecific background staining. Nonspecific binding was blocked with blocking solution (cat# TA-015-UB, ThermoFisher, CA, USA) for 7 min at room temperature in a humidity chamber. The sections were overnighted at +4 °C with primary antibody Epidermal Growth Factor (EGF) (cat# sc-374255, Santa Cruz Biotechnology Inc., Dallas, TX, USA, dilution ratio: 1/100 with distilled water). Sections were washed in phosphate-buffered saline (PBS) and biotinylated with a secondary antibody (cat# TP-015-BN, ThermoFisher, Fremont, CA, USA) for 14 min. Streptavidin–peroxidase (cat# TS-015-HR, ThermoFisher, Fremont, CA, USA) was dropped onto the section for 15 min, and EGF expression was visualized using chromogen Diaminobenzidine (DAB) (cat# TA-001-HCX, ThermoFisher, Fremont, CA, USA), where a brown color represents the expression of antibody of interest. After PBS washing, slides were counterstained with Gill III hematoxylin. Esophageal tissue sections were mounted and imaged under a Zeiss Imager A2 light microscope [31].

### 2.7. Statistical Analysis

IBM SPSS Statistics 25.0 (IBM Inc, Chicago, IL, USA) computer software was used for statistical analyses. Shapiro–Wilk tests were performed to evaluate the normality of the data. Continuous variables were presented as mean ± standard deviation (SD) or median (quartile 1–quartile 3). Within-group comparisons were analyzed by Analysis of Variance (ANOVA) (parametric) and post hoc Tukey tests, Kruskal Wallis test (non-parametric) and post hoc Dunn’s test. Significance level was considered as *p* < 0.05.

## 3. Results

### 3.1. The Merged Ellagic Acid PPI Network Analysis

**Figure 2:**
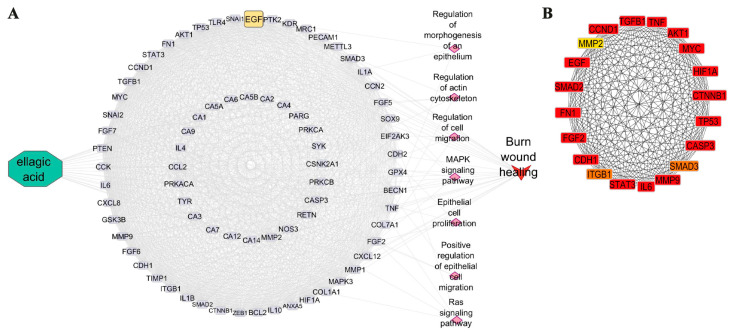
Ellagic acid associated PPI network and functional annotations. (**A**) Ellagic acid-target PPI network potential affected cellular events associated with wound healing. Diamonds (pink) represent functional annotations of EGF (yellow) associated with the essential wound-healing process. Arrow (red) indicates the Ellagic acid-targeted PPI network’s involvement in burn wound healing. (**B**) Twenty hub genes identified by MCC. The gradation from red to yellow signifies the higher ranking of hub genes. EGF (highlighted in red) has a high rank among 20 hub genes.

The merged PPI network of Ellagic acid and wound healing revealed that EGF was in the secondary level of Ellagic acid targets. The functional enrichment analysis of EGF in the different knowledgebase in Cytoscape software revealed that these genes are associated with critical processes involved in the regulation of morphogenesis of an epithelium (GO Biological Process), regulation of actin cytoskeleton (KEGG pathways and WikiPathways), regulation of cell migration (GO Biological Process), MAPK (KEGG pathways and WikiPathways) and Ras signaling pathway (KEGG pathways), epithelial cell proliferation (GO Biological Process), positive regulation of epithelial cell migration (GO Biological Process), which are pivotal regulators in the context of burn wound healing. Furthermore, a comprehensive functional annotation of all target proteins affected by Ellagic acid within the network demonstrated an influence on burn wound healing processes, as evidenced by insights gleaned from the WikiPathway knowledgebase (Figure 2A). The top 20 central genes in the PPI network were arranged as shown in Figure 2B, and EGF was among the high-ranking proteins in this PPI. EGF was found to hold a prominent position within the top 20 hub genes.,

### 3.2. Animal Weight

Animal weight was recorded before and at the end of the experiment in the control group, corrosive esophageal burn group (CEB) and CEB + Ellagic acid (EA) groups and is shown in Table 1. The post-experiment weights of animals in the CEB group were significantly decreased compared to their initial weight during the pre-experiment period. EA treatment alleviated the adverse symptoms of CEB injury in esophageal mucosa and led to a significant increase in the weight of animals at the end of the experiment in the CEB + EA group compared to their initial weights.

### 3.3. Malondialdehyde (MDA) Content

**Figure 3:**
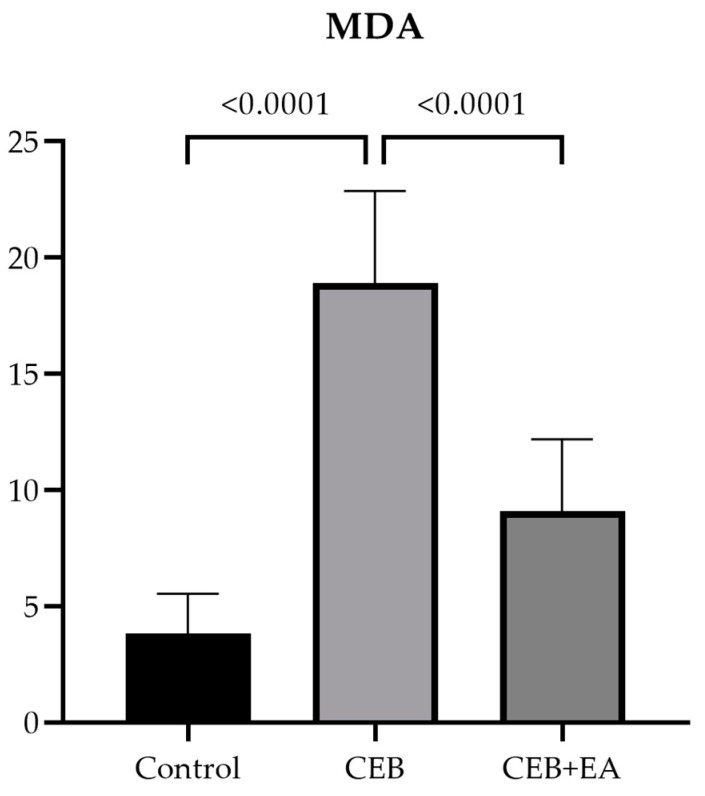
MDA content in experimental groups. (One-way ANOVA, post hoc Tukey).

MDA content of esophagus tissue was calculated at the end of the experiment and shown in Figure 3. EA treatment improved the MDA content after CEB injury. With its antioxidative property, EA helped the scavenging system of cells and favored the antioxidant enzymes after burn damage.

### 3.4. Histopathological Scoring

Semi-quantitative scoring of histopathological parameters of esophageal tissues in groups is shown in Table 2. The scores of SMI, MMD, TMCD, inflammation, fibrosis and necrosis were significantly increased in the CEB group; however, EA treatment restored these scores after burn injury. EA alleviated the adverse histopathological aspects of burn injury in the CEB + EA group.

**Figure 4:**
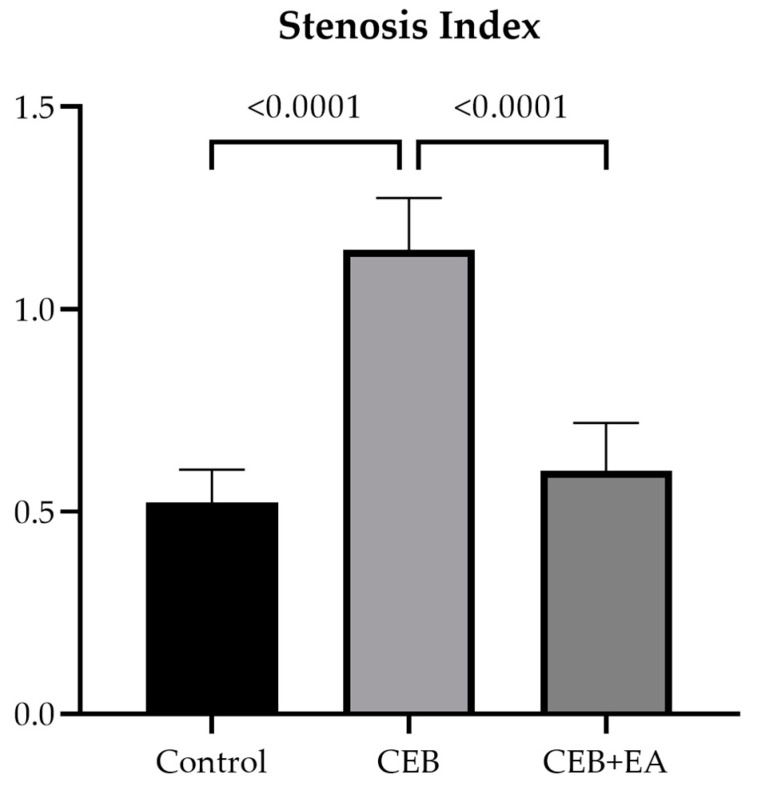
Stenosis index of experimental groups (one-way ANOVA, post hoc Tukey).

The stenosis index was calculated in esophageal sections of experimental groups and shown in Figure 4.. Burn injury increased the stenosis in the CEB group; however, EA reduced the stenosis index in the CEB + EA group due to its tissue-protective and -regenerative properties.

### 3.5. Histological Staining

**Figure 5:**
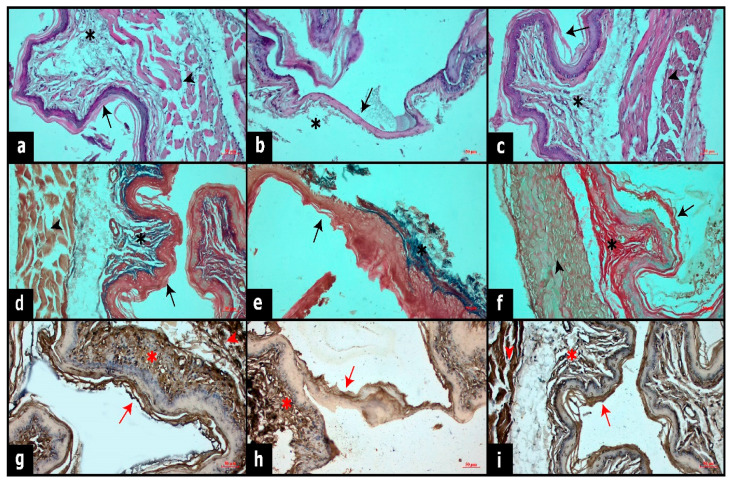
Histological staining of esophageal sections. (**a**–**c**): Hematoxylin and eosin staining of esophageal sections. (**a**). Control group. (**b**). CEB group. (**c**). CEB + EA group. (**d**–**f**): Masson trichrome staining of esophageal sections. d. Control group. (**e**). CEB group. (**f**). CEB + EA group. (**g**–**i**): EGF immune reactivities of esophageal sections. (**g**). Control group. (**h**). CEB group. (**i**). CEB + EA group. Arrow: epithelial layer with keratin formation; asterisk: connective tissue; arrowhead: muscular layer; Bar: 50 µm, Magnification: 20×.

The hematoxylin–eosin staining of esophageal sections is shown in Figure 5a–c. The control group showed a normal histological appearance in the esophageal tissue. The histological layers of the esophagus (tunica mucosa, tunica submucosa, muscular layer and tunica adventitia) were regular (Figure 5a). In the CEB group, the integrity of the mucosal layer was impaired due to burn damage, the amount of keratin and epithelium was reduced and the connective tissue degenerated (Figure 5b). In the CEB + EA group, the thickening of the keratin and mucosal layer, regeneration of the connective tissue and re-organization of the muscles were observed (Figure 5c). EA treatment improved the histopathology after burn injury in the mucosa and submucosa of the esophagus and provided a normal histological appearance with its tissue-protective properties.

The trichrome Masson staining of the esophageal sections is shown in Figure 5d-f to show fibrotic tissue. In the control group, the connective tissue was regular in the mucosal and submucosal layers (Figure 5d). However, fibrosis was observed in the CEB group. (Figure 5e). In the CEB + EA group, the proliferation of connective tissue and collagen fibers was restored (Figure 5f). EA treatment given after CEB prevents connective tissue degeneration and induces connective tissue cells, thus providing connective tissue regeneration.

The EGF immunostaining of the esophageal sections is shown in Figure 5g–i. In the control group, the EGF immune expression was intense in epithelial and connective tissue cells (Figure 5g). In the CEB group, a significant decrease in EGF expression was observed as a result of disruption of tissue integrity (Figure 5h). In the CEB + EA group, the EGF expression was increased in the mucosal and submucosal layers, compared to the CEB group (Figure 5i). EA played a role in tissue regeneration, involving cellular processes and proliferation in the epithelium. Additionally, it seems to contribute to tissue regeneration through the Epidermal Growth Factor (EGF) signaling pathway.

## 4. Discussion

The ingestion of alkaline corrosive substances causes chemical burns in the upper gastrointestinal tract, especially the esophagus, due to their high viscosity and pH level [32]. Fibrous tissue is the cause of the formation of the esophageal stricture in the chronic period [5,26,27]. To prevent stricture formation and treat corrosive esophageal burn (CEB) injury, ketotifen [33], tamoxifen [34], Ankaferd Blood Stopper [35], carvacrol [36], hypericum perforatum oil [37], contractubex [30], ethyl pyruvate, N-acetyl cysteine [38], platelet-rich plasma [39], alpha lipoic [40], bromelain [41], hesperidin [42] and mitomycin C [43] were studied. Due to its antioxidant, anti-inflammatory, antifibrotic [44], antitumoral [45], antiviral [46] and antidiabetic [47] effects, this study investigated the efficacy of Ellagic acid (EA) for the treatment of CEB injury, as previous experimental studies explored various therapeutic agents. However, to the best of our knowledge, none of these agents demonstrated conclusive effectiveness in the treatment of CEB injury. Furthermore, potential of EA in this regard remains unexplored in the existing literature. Therefore, my research aims to fill this gap by evaluating the therapeutic potential of EA in the context of CEB injury. EA scavenges free radicals and mitigates oxidative stress, creating a microenvironment for tissue repair and regeneration, which may enhance the epidermal growth factor (EGF)-mediated signaling [11]. Considering these, this study evaluates the effects of EA on esophageal burn via the EGF pathway.

Stricture formation in CEB injury is followed by dysphagia and may cause malnutrition. Anayurt et al. [42] recorded the body weights of animals after an experimental CEB model induced by 20% NaOH. At the end of the 28-day experiment, body weights were found to be significantly lower in the CEB group compared to the control group. In this study, the initial weight of animals was compared with the final weight of the animals within the same group. The initial weights of animals in the CEB group were significantly decreased after burn injury. The initial weight of animals in the CEB + EA group was decreased at the end of the experiment; however, EA treatment significantly prevented the significant decline of weight in animals. The decrease in animal weights in the burn group is likely a consequence of the physiological stress and trauma induced by the burn injury. Burn injuries can lead to metabolic changes, increased energy expenditure and tissue damage, all of which can contribute to weight loss. The weight loss of rats in the EA-treated group may show the contribution of EA to the general health and well-being of these animals and their recovery. This could be attributed to EA’s ability to counteract the detrimental effects of burn injuries, fostering a more favorable environment for recovery.

Free oxygen radicals cause tissue damage by increasing lipid peroxidation. Malondialdehyde (MDA) is the end product of lipid peroxidation and causes cell damage by disrupting the cell membrane structure [48]. In the CEB model created with 15% NaOH, the MDA level increased significantly in the CEB group compared to the control group [40]. Another study showed that the antioxidant activity of EA reduced the MDA level significantly in the mucositis + EA group compared to the mucositis group [41]. Our study is in line with a previous study and showed that the MDA level increased in the CEB group; however, EA administration reduced MDA content significantly after CEB. The decrease in MDA levels in the EA-treated group suggests that EA acts as a scavenger of free radicals, preventing lipid peroxidation and ultimately contributing to a reduction in MDA levels.

The scoring of histopathological parameters is used as a semi-quantitative method to determine the level of tissue damage in CEB injury and to predict stricture formation [42,49]. Similarly, the esophageal stenosis index is used as an indicator of stricture formation [50]. Akay et al. [37] found that the stenosis index in the CEB group induced by 10% NaOH increased significantly compared to the control group. In 30% of NaOH-induced CEB injuries, damage to the muscularis mucosa was significantly increased in the CEB group compared to the control group. The increase in submucosal collagen deposition was significantly higher in the CEB group compared to the control group. In another study, the stenosis index was found to be significantly higher in the CEB group compared to the control [26]. This study found that esophageal burns caused adverse histopathological alterations such as mucosal damage, increased collagen deposition and a damaged muscular layer in the CEB group. Inflammation, fibrosis and necrosis were increased in the CEB group. In the EA-treated group, EA prevented the further progress of these parameters, suggesting EA has protective, anti-inflammatory positive effects on preserving the integrity of the mucosal muscular layer, potentially inhibiting excessive collagen deposition and promoting tissue regeneration and repair.

Wound healing comprises stages like hemostasis, inflammation, angiogenesis, growth, re-epithelialization, remodeling and the regulation of protein kinases. These stages follow a sequential but overlapping pattern, complicating the repair processes [51]. Network analysis provides support for the notion that Ellagic acid targets EGF, which plays essential roles in crucial cellular events associated with wound healing. Furthermore, the PPI network analysis indicates that Ellagic acid is a suitable compound for investigating burn wound healing, as its targeted proteins are involved in this process, potentially influencing healing outcomes in a significant manner. EGF was identified as a central player in this protein–protein interaction network and is integral to the wound healing process due to its involvement in activities like proliferation, migration, morphogenesis regulation, cytoskeleton maintenance, as well as the regulation of MAPK activity and Ras signaling [52,53,54].

The roles of many natural molecules were investigated in experimental CEB injury. Sun et al. [55] studied xenograft tumors in the esophagus and found that gallic acid treatment reduced the volume of the esophageal carcinoma and Ki67 expression, a cell proliferation marker, via the Hippo signal pathway. Özden et al. [56] investigated the roles of resveratrol in the corrosive esophagitis model and found that the total antioxidant status was increased, and the total oxidant status and levels of inflammation markers (IL1, IL6 and Tnfα) were decreased after resveratrol administration to rats. Another study showed that Carvacrol significantly restored the histopathology of CEB injury, induced re-epithelization, reduced apoptosis rate via downregulating the caspase-3 and reduced fibrosis [36]. The roles of vitamin E and vitamin C were investigated in corrosive esophageal burn injury in rats. The results showed that vitamin-treated groups significantly reduced the MDA levels and prevented collagen deposition in the esophageal layers after CEB injury [57]. The effects of melanin were also shown in CEB injury. Chornenka et al. [58] found that melanin decreased the MDA content and favored the functions of antioxidant enzymes.

Preclinical studies also support the role of EA and EGF on wound healing. Lin et al. [59] studied the role of EA in the suppression of cardiac fibroblasts in myocardial infarction and found that EA has an anti-fibrotic effect via downregulating collagen I and III in rat cardiac fibroblasts. The authors claim that EA has therapeutic effects against fibrosis. Lu et al. [60] studied the effects of EA on wound healing in vitro and in vivo. They showed that EA induced proliferation of epidermal cells, effectively promoted cell growth and decreased the wound area in mice. EGF accelerates the wound-healing process by inducing keratinocyte proliferation and migration in wound healing [61] and the proliferation of endothelial cells and fibroblasts [62]. Koltuksuz et al. [63] stated that EGF treatment was useful in preventing increased collagen accumulation and stricture development in the CEB group induced with 50% NaOH. In a similar study, the role of EGF was investigated in CEB, and the study revealed that collagen synthesis was significantly higher in the EGF-treated group than in collagen synthesis of the untreated group on the 5th postoperative day with a lower esophageal stenosis index in the CEB + EGF group [64]. In this study, compared to the control group, EGF immune activity was decreased in epithelial and connective tissue cells in the CEB group, indicating tissue damage caused by the CEB. In the CEB + EA group, the EGF expression was elevated intensely in the mucosal and submucosal layers. The increased EGF expression in the CEB + EA group suggests that EA treatment has a positive impact on restoring EGF levels. This restoration of EGF expression in the treated group may indicate that EA helps in preserving or enhancing the signaling pathways associated with tissue repair and regeneration.

### Limitations

The study utilized a relatively small sample size; however, a larger sample size would provide more statistical power and enhance the reliability of the findings. Animal models may not fully replicate the complex pathophysiology and clinical manifestations of CEB injury in humans. The experimental period of 28 days may be insufficient to fully evaluate the long-term effects of EA treatment on wound healing and esophageal tissue regeneration. Besides MDA content and histological staining, additional outcome measures, such as markers of inflammation, angiogenesis and extracellular matrix remodeling, could provide a more comprehensive understanding of the mechanisms underlying EA-mediated wound healing. Further mechanistic studies are needed to unravel the precise pathways through which EA exerts its therapeutic effects in CEB injury.

## 5. Conclusions

EA treatment contributes to overall animal health, indicating a positive impact on recovery. With its antioxidative property, EA enhanced the scavenging system and favored antioxidant enzymes post-burn damage. EA treatment effectively demonstrated histopathological counteracts the detrimental effects of CEB, restoring normal tissue architecture, reducing collagen increase and ameliorating inflammation, fibrosis and necrosis. EA contributed to increased EGF expression, further supporting its role in cellular processes crucial for tissue regeneration. Ellagic acid emerges as a promising therapeutic agent; however, further comprehensive preclinical and clinical studies are needed.

## Figures and Tables

**Table 1:** Animal’s weight pre- and post-experiment.

Groups	Pre-Experiment (Mean + SD, g)	Post-Experiment (Mean + SD, g)	Pairwise Comparison
Control	263 ± 9.9	262 ± 9.1	*p* = 0.78
CEB	264 ± 4.6	217 ± 12	*p* < 0.0001
CEB + EA	260 ± 8.1	242 ± 6.6	*p* = 0.0012

Test: Paired t test, SD: standard deviation, g: gram, CEB: corrosive esophageal burn, EA: Ellagic acid.

**Table 2:** Scoring of histopathological parameters of esophageal tissue in control, CEB and CEB + EA.

Groups	Control Median (Q1–Q3)	CEB Median (Q1–Q3)	CEB + EA Median (Q1–Q3)
SMI	0.00 (0.00–0.00)	1.00 (1.00–2.00) *	0.50 (0.00–1.00) **
MMD	0.00 (0.00–0.00)	1.00 (1.00–1.00) *	0.00 (0.00–1.00) **
TMCD	0.00 (0.00–0.00)	1.00 (1.00–2.00) *	1.00 (0.00–1.00) **
Inflammation	0.00 (0.00–0.00)	3.00 (3.00–3.00) *	0.00 (0.00–1.00) **
Fibrosis	0.00 (0.00–0.00)	2.00 (2.00–3.00) *	0.00 (0.00–1.00) **
Necrosis	0.00 (0.00–0.00)	1.00 (1.00–1.00) *	0.00 (0.00–0.00) **

* Control vs. CEB, ** CEB vs. CEB + EA, test: Kruskal–Wallace, post hoc Dunn’s test, *p* < 0.0001; SMI: submucosal collage increase, MMD: mucosal muscular layer damage, TMCD: collagen increase and damage in muscular layer.

## Data Availability

All generated data were used in this study.
